# The WOCA negative pressure wound therapy device designed for low resource settings^☆^

**DOI:** 10.1016/j.ohx.2024.e00620

**Published:** 2024-12-20

**Authors:** Arjan J. Knulst, Salome Berger, Jorijn van den Boom, Inge Bosch, Noa Nicolai, Suraj Maharjan, Eileen Raaijmakers, Chang-Lung Tsai, Lisa van de Weerd, Jenny Dankelman, Jan-Carel Diehl

**Affiliations:** a Department of Biomechanical Engineering, Delft University of Technology, Delft, the Netherlands; b Biomedical Engineering Department, Green Pastures Hospital and Rehabilitation Center, International Nepal Fellowship, Pokhara, Nepal; c Industrial Design Engineering, Delft University of Technology, Delft, the Netherlands; d Shining Hospital Surkhet, International Nepal Fellowship, Birendranagar, Nepal; e Reconstructive Surgery Department, Green Pastures Hospital and Rehabilitation Center, International Nepal Fellowship, Pokhara, Nepal

**Keywords:** Medical device, Negative pressure wound therapy, Vacuum-assisted closure therapy, Wound care, Low-cost, Low-resource setting

## Abstract

Negative Pressure Wound Therapy (NPWT) is a treatment that promotes healing of chronic wounds. Despite high prevalence of chronic wounds in Low- and Middle-Income Countries (LMICs), NPWT devices are not available nor affordable. This study aims to improve chronic wound care in LMICs by presenting the Wound Care (WOCA) system, designed for building, testing and use in LMICs. Design requirements were formulated using input from literature, ISO standards, and wound care experts. The WOCA design was developed to provide safe, portable, user-friendly and affordable NPWT to patients in LMICs. The design features an adjustable operating pressure ranging from −75 to −125 mmHg, a battery for portability, a 300 ml canister, overflow protection, and system state alarms. An Arduino controls the pressure and monitors the system state. Three prototypes were developed and built in Nepal, and their performance was evaluated. Pressure control was 125 ± 10 % mmHg, internal leakage was 7.5 ± 4.3 mmHg/min, reserve capacity was 189 ± 16.9 ml/min, and overflow protection and alarm systems were effectively working. Prototype cost was approximately 280 USD. The WOCA demonstrates to be a locally producible NPWT device that can safely generate a stable vacuum. Future research will include clinical trials situated in LMICs.

## Graphical abstract

**Figure:**
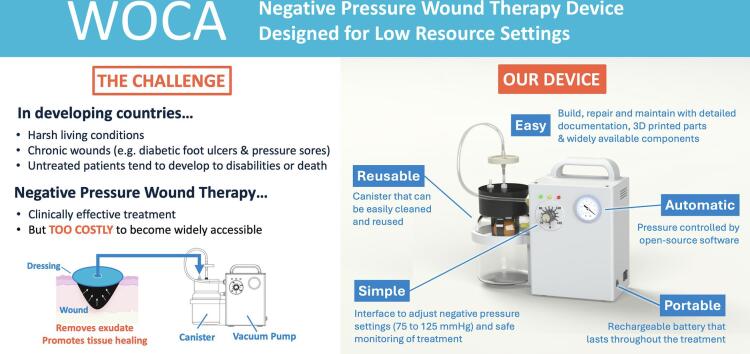

## Specifications table

**Table:** 

Hardware name	WOCA Wound Pump
Subject area	• Biomedical Engineering
	• Open source and low-cost alternatives to existing devices
	• Low-resource Contexts
Hardware type	• Medical Device
	• Negative Pressure Wound Therapy Pump
	• Vacuum Assisted Wound Closure Pump
Closest commercial analog	3 M™ ActiV.A.C.™ Therapy Unit
Open source license	Creative Commons Attribution 4.0 International license
Cost of hardware	Approximately 280 Euros
Source file repository	https://doi.org/10.17632/r95wgtmffn

## 1 Hardware in context

### 1.1 Highlights

Challenges:

• Harsh living conditions contribute to high prevalence of chronic wounds in developing countries.
• While negative pressure wound therapy is clinically effective, its high cost limits its accessibility.

Key features of our device:

• **Automatic** pressure control with open-source software.
• **Portable** design with a battery that lasts at least one treatment cycle (3–5 days).
• **Simple** interface to adjust negative pressure settings (75 to 125 mmHg) and safe treatment monitoring.
• **Easy** to build, repair, and maintain with detailed documentation, 3D printed parts, and widely available components.
• **Reusable** canister that can be easily cleaned and reused.
• **Affordable** design having an approximate component cost of 280 USD.

### 1.2 Background

Wound management remains a serious issue in developing countries or Low- and Middle-Income Countries (LMICs). Poor access to healthcare and challenging living conditions tend to contribute to the high prevalence of chronic wounds in these regions [1], [2]. Chronic wounds such as diabetic foot ulcers and pressures sores are everyday occurrence for many people living in LMICs. Without adequate treatment, such wounds may lead to permanent disabilities or even death [2].

Negative Pressure Wound Therapy (NPWT), also known as Vacuum Assisted Closure (VAC), is a versatile and effective treatment that aids the healing of chronic wounds. It was first introduced by Argenta and Morykwas in 1997 [3] and has since gained widespread popularity as an effective treatment with numerous clinically proven benefits [3], [4], [5], [6], [7]. Wounds treated with NPWT typically heal faster [3], require fewer dressing changes [5], and involve less frequent surgical interventions [4]. Consequently, NPWT reduces hospital stays for patients and alleviates the workload of medical staff [5], [6]. NPWT also enhances patient comfort by improving mobility [4], [5] and ensuring hygienic wound closure [3], [4]. The combined effects of suction and pressure in NPWT activate a cascade of physiological responses that collectively promote wound healing [8]. These effects include:

• Stabilization of the wound environment;
• reduction of wound edema;
• decrease in bacterial load;
• enhanced tissue perfusion and angiogenesis.

By creating a bacteria-free and oxygen-rich environment, NPWT fosters the formation of granulation tissue, a crucial precursor to wound closure. The rate of granulation tissue formation, often used as a marker of healing success in clinical trials, is significantly accelerated under NPWT [9].

Treatment for NPWT requires a vacuum device that is connected to a sealed vacuum dressing. Although it has been suggested that NPWT can be more economical in the long term compared to conventional wound treatment [10], it requires a large initial investment due to the high cost of the device. NPWT-device prices range from 7500 to 12,000 USD and dressing price range from 30 to 50 USD [11]. These dressing kits are pre-sized and meant for single-use, therefore a continuous supply and sufficient stock of dressings is required. NPWT devices are often leased through monthly payment which includes services for maintenance and repair. Note that prices are estimated, and the actual prices cannot be retrieved as vendors do not share information on their arrangements with customers.

The average treatment costs for NPWT in a regular hospital are estimated around 116 USD per patient per day, which is approximately 5 % higher compared to standard treatment [12]. Another study on Medicare Home Health Patients have shown that average cost per treatment was 899 USD for traditional devices and 1624 USD for disposable (single-use) devices [13].

NPWT device industry has traditionally been dominated by Western companies [11]. The American company KCI, now part of 3 M, sponsored the first studies on vacuum wound therapy in 1997. KCI promptly patented the technology, securing its position as the sole manufacturer of NPWT devices for a decade. During this time, their traditional V.A.C.® System was the only NPWT device available. It was only after the patent expired that other companies were able to enter the market.

Currently, two types of NPWT systems are available: traditional and single-use. Traditional systems feature a canister for collecting fluid from the wound and offer adjustable pressure settings, with both continuous and intermittent modes of operation. These devices are often powered by electricity and are primarily used in inpatient settings. In contrast, single-use NPWT systems, such as Smith & Nephew’s PICO, are canister-free and manage wound exudate through evaporation via the outer layer of the dressing. These devices deliver continuous, non-adjustable pressure, are battery-powered, and are more commonly used in outpatient care. However, NPWT systems that are currently on the market are not suitable for low-resource settings since these products:

• Are expensive (>7.500 USD) or meant for single-use;
• rely on specific dressings;
• are poorly accessible because mostly are shipped from the US or Europe;
• use advanced technology and therefore require technical support;
• are complex to use and therefore require a certain level of training.

**Fig. 1:**
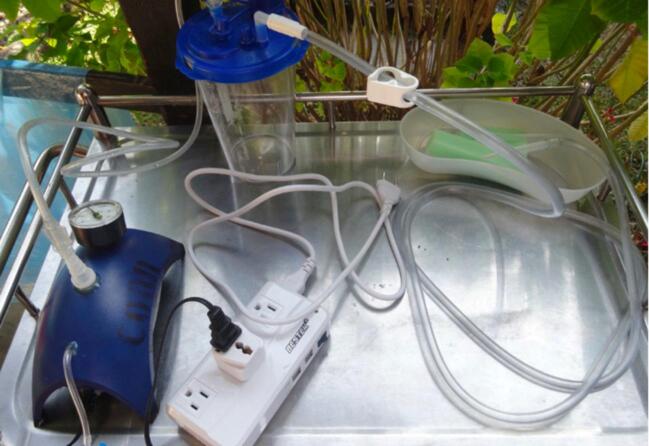
Setup of the converted aquarium pump (AquaVAC) used in Green Pastures Hospital. (For interpretation of the references to colour in this figure legend, the reader is referred to the web version of this article.)

In the Green Pastures Hospital originally a converted aquarium pump AquaVAC (Fig. 1) was used. Though effective in terms of wound healing [14], various factors inherent to the device design limit their usability and safety. The device has limited suction performance, and has lack of pressure control and safety alarms. Tripping risks exist because of the many separate connected components: pump, canister, tubes, power adaptor (110 to 230 V) and power cords. The lack of battery power limits patient mobility. And as these devices are generally imported from abroad poor repairability or replacement exist. These limitations increase patient risk, reduce user friendliness, and limit sustainability of the device.

### 1.3 Project aim and design

Under the above mentioned context a project was initiated to develop a device for suitable use in LMICs. The aim was to: “Develop a low-cost, portable and safe NPWT system that uses standard and widely available components and consumables that can be assembled in LMICs”.

**Fig. 2:**
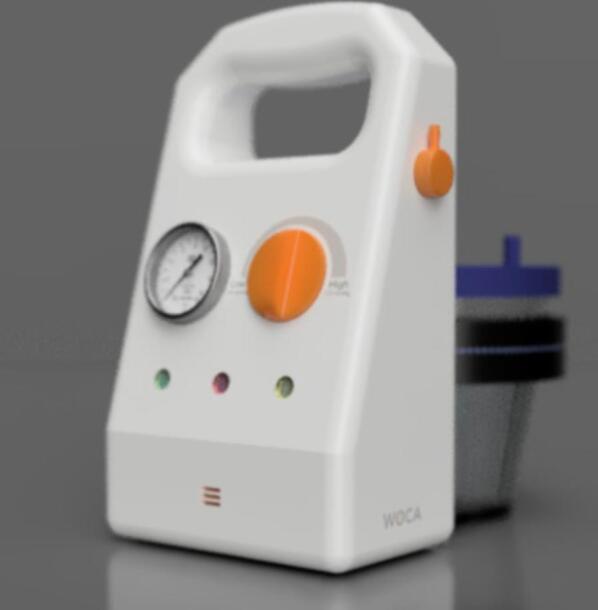
Conceptual design of the WOCA Pump [11].

An initial conceptual design of the Wound Care (WOCA) Pump (Fig. 2) was made [11]. Based on this concept, a functional prototype was constructed, featuring a 3D printed housing and an Arduino microcontroller [15]. Current products that are easy to use are designed for single-use, while traditional options are often complex and less accessible. The prototype aims to address a market gap by offering a simple, reusable NPWT device. The WOCA design integrates key functionality like adjustable pressure control (−75 to −125 mmHg, controlled within ± 10 % margin), pressure and time based alarm limits to ensure patient safety, a rechargeable battery that can last a full treatment cycle (12 V 6600 mAh), and an integrated canister (300 ml usable fluid capacity). The WOCA design eliminates power-intensive features such as LCD touchscreens, and reduces complexity by allowing continuous pressure treatment only. By reducing complexity and focusing on key-functionality the device provides a basic yet portable, functional, safe, and cost-effective NPWT solution.

A small series of three prototypes was constructed in the INF Green Pastures Hospital using locally sourced components to verify and validate the concept. The design was verified against its technical specifications and validated against clinical user requirements.

In this document, the latest developments of the WOCA Pump will be presented. A detailed technical documentation of the latest design will be shared, in the hope of narrowing gaps and decreasing barriers to healthcare in LMICs.

### 1.4 Limitations

Further clinical validation is planned in a clinical trial to evaluate its effectiveness in a clinical setting and compare it to documented effectiveness of commercial devices. This study is currently in preparation.

Further testing on durability of components and materials, and repairability is required. No reliability testing until failure was done yet, as these prototypes are intended to have a role in the future clinical study that is being prepared. These durability tests will also test the repairability when breakdowns occur. As parts were sourced locally and assembled locally the repairability should not be a problem, however, it needs to be validated in the near future.

## 2 Hardware description

### 2.1 Design

**Fig. 3:**
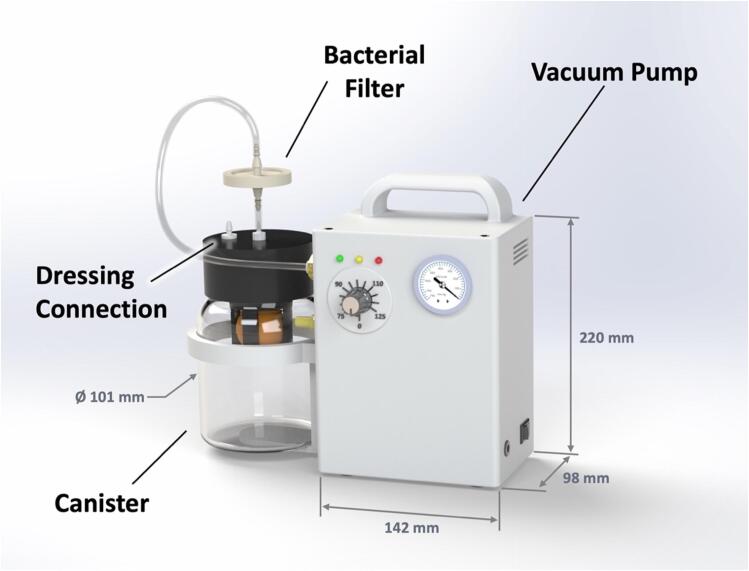
The WOCA Pump consists of a vacuum pump, bacterial filter, and canister.

**Fig. 4:**
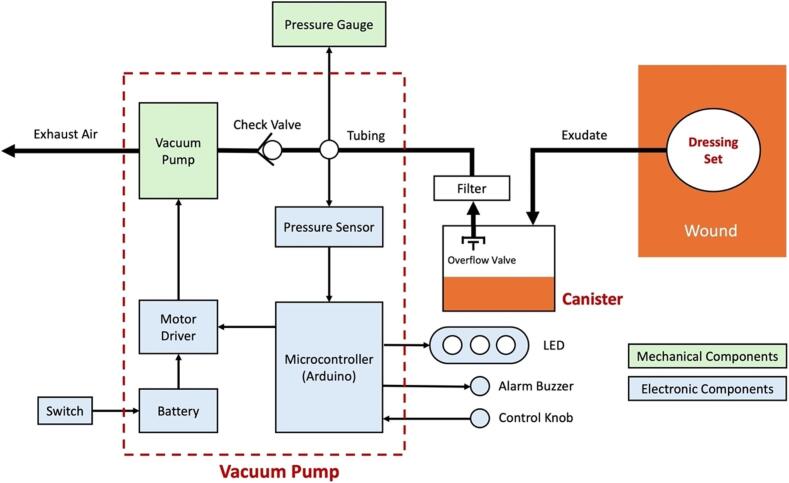
System Block Diagram of WOCA Pump.

The WOCA Pump (Fig. 3) is a medical device designed as an easy-to-use system that can be easily modified and made compatible to different wound closure dressing pads. It includes a vacuum pump and a canister. The vacuum pump is connected to the canister and creates negative pressure. Dressing pads applied to the wound can be connected to the canister to apply vacuum to the wound and to remove exudate. A block diagram of how the WOCA pump functions is shown in Fig. 4.

**Fig. 5:**
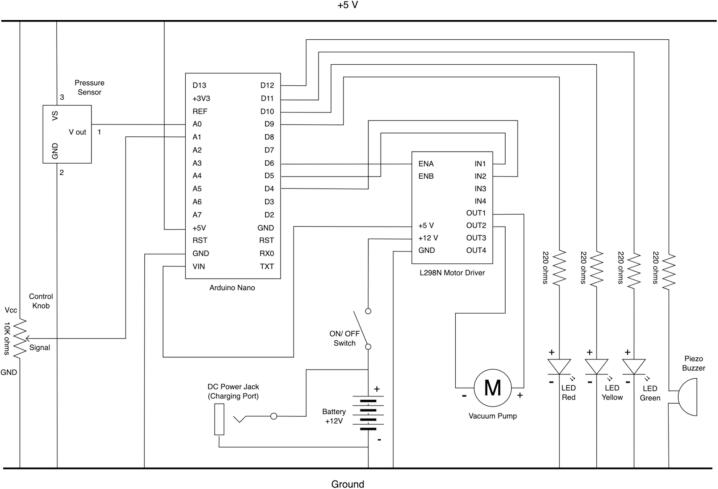
Schematic of the electrical circuit in the WOCA Vacuum Pump.

The hardware is easy to manufacture and assemble. It relies on commonly available electronic, mechanical and additively manufactured parts. To control the system various hardware components are used. A schematic of the electrical circuit design is shown in Fig. 5. These include a microcontroller (Arduino Nano), DC motor driver (L298N), and a pressure sensor (MPX5050DP). A simple interface that includes a pressure gauge, LEDs and alarm buzzer provides necessary information to the user. Controls include a power switch and control knob for adjusting pressure required for the treatment. Power is provided by a 12 V 6800 mAh lithium battery that can be recharged through a DC charger (12.6 V 2A) delivered with the battery. The use of wire to board connectors eases the (dis)assembly process. The open-source Arduino environment provides an easy platform for further modification of the software. The canister is also designed to be reused and cleaned. Parts can be easily disassembled, cleaned, reassembled, and then reused. The only consumable is the canister filter that needs to be periodically replaced.

### 2.2 Control software and safety alarms

**Fig. 6:**
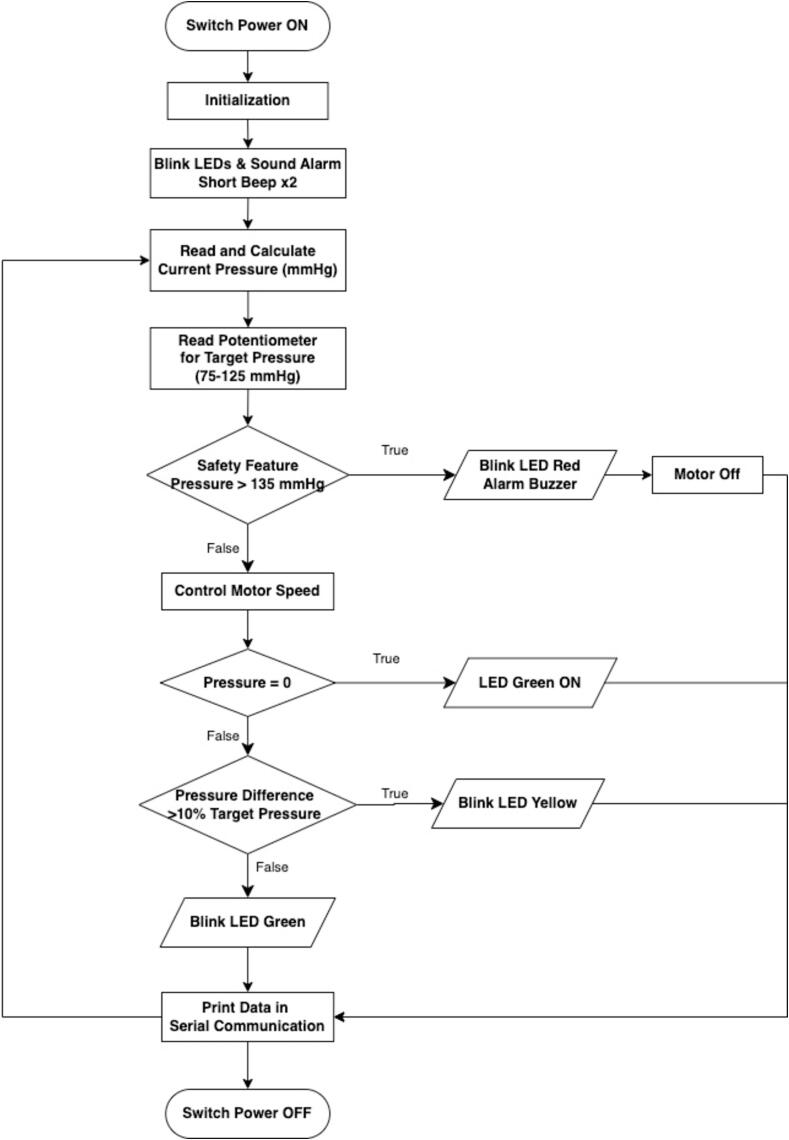
Flowchart of Arduino control software of the WOCA Pump.

The software (Arduino IDE) is designed to monitor and regulate pressure with integrated safety features. The flowchart for the program is shown in Fig. 6. The process begins with an initialization phase, during which the system blinks LEDs and emits two short beeps through an alarm buzzer, signaling readiness for operation. These indicators also serve as diagnostic tools, allowing users to verify that the device's components are functioning correctly.

Once initialized, the system enters a continuous monitoring loop, sampling pressure values every 25 ms. These values are averaged over the last 40 measurements to enhance accuracy and stability. Users can set the target pressure within a range of 75–125 mmHg using a control knob connected to a potentiometer. A built-in safety mechanism ensures that the pressure never exceeds 135 mmHg. If this safety threshold is breached, the system activates a red LED, sounds the alarm, and automatically shuts off the motor to prevent any risk to the patient.

Under normal operating conditions, the motor adjusts dynamically to maintain pressure within ± 10 % of the target value. The motor activates when the pressure falls below the target and stops once the target is exceeded. The system continuously evaluates the pressure status to ensure reliability. If the device is powered on but no vacuum pressure is detected, a green LED illuminates, indicating that the device is powered on. If the pressure deviates by more than 10 % from the target, a yellow LED blinks as a warning. When the pressure stabilizes within the acceptable range, a green LED blinks to confirm normal operation.

The system also supports real-time data transmission via serial communication for development and troubleshooting. When connected to a computer through USB, users can monitor live pressure readings and motor performance for analysis and diagnostics, enabling efficient data logging and troubleshooting.

To prevent unwanted reprogramming of the device, lock and fuse bits on Arduino microcontrollers can be configured to secure the firmware. Fuse bits can be set to disable programming via the boot loader, and lock bits can restrict further changes to the fuse configuration, protecting the microcontroller's memory from unauthorized access or modification. For additional physical protection, the USB port can be permanently sealed with strong adhesive or physically removed, making it impossible to access the microcontroller without disassembly. These combined measures ensure the integrity of the firmware and maintain safe, reliable device operation.

### 2.3 Hardware performance

**Fig. 7:**
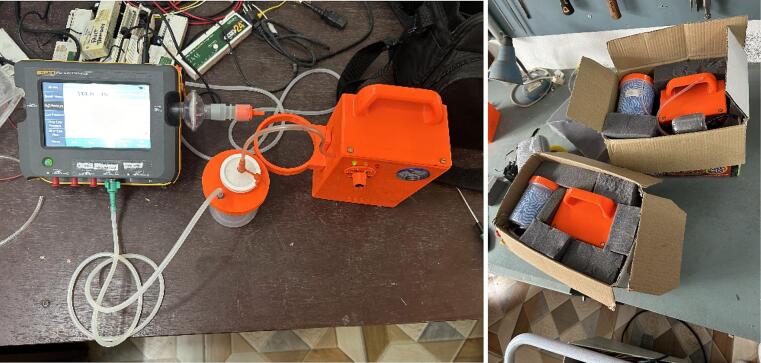
Testing of the WOCA prototype in Nepal. (Left) Pressure and leak tests with a gas flow analyzer. (Right) Packaging of the prototype for transportation test.

Three prototypes were constructed in Nepal to evaluate the performance of the design [16]. The pressure was set at 125 mmHg and monitored continuously for 30 min using a gas flow analyzer (Fluke V900A) (Fig. 7a). Results indicated that all three prototypes maintained pressure within the acceptable ± 10 % range over these 30 min. Internal system leakage (Fig. 7a) of these 3 devices (when vacuum pump switched off) were 2.2, 12.8, and 7.5 (7.5 ± 4.3) mmHg/min for prototype 1, 2 and 3, respectively. This test includes all internal connections and the internal check-valve that prevents backflow through the motor when switched off.

The prototypes were also tested on their reserve capacity. This is the maximum allowable external leak rate where the WOCA still maintains its pressure set point. Air leak was introduced at the patient tube to simulate leakage and slowly increased until the system could no longer maintain its set pressure. The measured reserve capacities were 200, 165, and 202 (189 ± 16.9) ml/min.

Battery life was not fully tested, but preliminary results showed that all prototypes could sustain the target pressure (125 mmHg) for at least 8 h. Theoretically, the 6600 mAh 12 V battery, connected to a 12 V vacuum pump drawing 1A when operating, would last 6.6 h of continuous vacuum motor use. However, the pump is not running continuous. During a 5-minute test, the total motor activation time was 2050 ms, equating to about 0.68 % of the duration. This gives a total of 40 days of intermittent use. As treatment is expected to be 5 days the battery should have sufficient capacity for a single treatment on a single battery charge.

However, additional testing is needed to evaluate the cumulative power consumption of other components, including LED indicators, the motor driver, and microcontrollers, as these factors may significantly affect overall battery performance. Additionally, the quality of the wound dressing may have a significant impact on motor operation. Testing thus far has been conducted without actual dressings. Poorly dressed wounds could result in greater air leakage, necessitating more frequent motor activation and further reducing battery life.

Additionally, the prototypes were subjected to transportation testing on challenging road conditions in Nepal. Each device was packed in a cardboard box with foam padding (Fig. 7b) and transported for 8 h in a 4x4 vehicle over a mix of paved and unpaved roads to a different hospital. The prototypes showed no visible damage or operational issues after transportation.

## 3 Design files summary

This section features a list of all the files needed to reproduce the WOCA Pump (Table 1). The bill of materials (Designator: BOM) of the vacuum pump and canister are provided separately in Excel file and pdf format. The assembly (Designator: ASM) and part (Designator: MFG) files are constructed in Solidworks 2024. In case of the need for further modification, a software version of at least 2024 may be needed. The STL files of the parts that needed to be 3D printed are also provided in addition to the Solidworks part file. The software code (Designator: SW) used to control the microcontroller is provided in INO file format that can be opened and modified in Arduino IDE.

**Table 1:** WOCA Pump design files.

**Designator**	**Design file name**	**File type**	**Open-source License**	**Location of the file**
BOM01	WOCA Vacuum Pump	.xlsx, pdf	CC 4.0	Specifications Table: Source file repository
BOM02	WOCA Canister	.xlsx, pdf		
ASM01	Vacuum Pump Assembly	.sldasm		
ASM02	Canister Assembly	.sldasm		
MFG01	Front Housing	.stl,.sldprt		
MFG02	Back Housing	.stl,.sldprt		
MFG03	Canister Holder	.stl,.sldprt		
MFG04	Hole Grid	.stl,.sldprt		
MFG05	Canister Lid	.stl,.sldprt		
MFG06	Ball Cage	.stl,.sldprt		
MFG07	Piston	.stl,.sldprt		
MFG08	Pressure Setting Plate	.stl,.sldprt		
SW01	WOCA Arduino Code	.ino		

## 4 Bill of materials summary

The complete bill of materials is provided online in the design files folder. Additional information such as specifications, manufacturer, model, and links to the sources are listed in detail. The bill of materials is listed separately for the vacuum pump and canister in Tables 2 and 3. All components and consumables such as filaments and wires required to build one WOCA pump are listed. 3D printed parts are manufactured in-house, which has no additional costs besides material cost. The cost of the used filament is included in the tables below.

**Table 2:** Bill of materials of the WOCA Vacuum Pump.

**Designator**	**Component**	**Number**	**Cost per unit- €**	**Total cost − €**	**Source of materials**	**Material type**
Manufactured Parts						
MFG01	Housing, Front	1 pcs	N/A	N/A	Delft University of Technology Workshop	PET-G
MFG02	Housing, Back	1 pcs	N/A	N/A		PET-G
MFG03	Canister Holder	1 pcs	N/A	N/A		PET-G
MFG04	Hole Grid	1 pcs	N/A	N/A		PET-G
MFG08	Pressure Setting Plate	1 pcs	N/A	N/A		PET-G
Procured Mechanical Parts						
ME01	Vacuum Pump	1 pcs	17.84	17.84	Mouser NL	Metal
ME02	Pressure Gauge	1 pcs	9.74	9.74	Dominga IT	Metal
ME03	Connector, Bulkhead	1 pcs	3.61	3.61	Distrelec NL	Brass
ME04	Connector, T	2 pcs	3.24	6.48	Distrelec NL	Polymer
ME05	Connector, Elbow Female	1 pcs	2.67	2.67	RS NL	Brass
ME06	Connector, DiffOD	1 pcs	3.09	3.09	Distrelec NL	Polymer
ME07	Connector, Hose	1 pcs	0.68	0.68	Distrelec NL	Brass
ME08	Check Valve	1 pcs	8.90	8.90	Distrelec NL	Polymer
ME09	Silencer	1 pcs	1.81	1.81	Distrelec NL	Brass
ME10	Tubes, OD 6 mm	2 *m*	0.51	1.02	Distrelec NL	PU
ME11	Tubes, OD 8 mm	1 *m*	0.72	0.72	Distrelec NL	PU
ME12	Bolt, M4x16mm	2 pcs	0.11	0.22	Distrelec NL	Stainless Steel
ME13	Bolt, M4x10mm	5 pcs	0.08	0.40	Distrelec NL	Stainless Steel
ME14	Bolt, M3x20mm	2 pcs	0.07	0.14	Distrelec NL	Stainless Steel
ME15	Bolt, M3x16mm	8 pcs	0.06	0.48	Distrelec NL	Stainless Steel
ME16	Bolt, M3x6mm	1 pcs	0.06	0.06	Distrelec NL	Stainless Steel
ME17	Nut, M4	7 pcs	0.04	0.28	Distrelec NL	Stainless Steel
ME18	Nut, M3	10 pcs	0.03	0.30	Distrelec NL	Polymer
ME19	Cable Ties	1 pack	3.37	3.37	Distrelec NL	Polymer
ME20	Rubber Feet	4 pcs	0.12	0.48	Distrelec NL	NBR
ME21	Thread Seal Tape	1 roll	1.30	1.30	Distrelec NL	PTFE
ME22	Foam Tape	1 roll	5.49	5.49	Amazon NL	Polymer
ME23	3D Printer Filament	866 g	0.05	47.63	RS NL	PET-G
Procured Electronic Parts						
EE01	Microcontroller	1 pcs	23.16	23.16	Mouser NL	Electronics
EE02	Motor Driver	1 pcs	7.11	7.11	Reichelt	Electronics
EE03	Pressure Sensor	1 pcs	24.00	24.00	Mouser NL	Electronics
EE04	Potentiometer	1 pcs	0.84	0.84	Mouser NL	Electronics
EE05	Control Knob	1 pcs	0.46	0.46	Mouser NL	Polymer
EE06	Resistor, 220 O	4 pcs	0.09	0.36	Mouser NL	Electronics
EE07	LED Red	1 pcs	0.36	0.36	Mouser NL	Electronics
EE08	LED Green	1 pcs	0.36	0.36	Mouser NL	Electronics
EE09	LED Yellow	1 pcs	0.36	0.36	Mouser NL	Electronics
EE10	Piezo Buzzer	1 pcs	1.34	1.34	RS NL	Electronics
EE11	Lithium Battery	1 pcs	37.15	37.15	LedstripKoning	Electronics
EE12	Prototype Board	1 pcs	5.50	5.50	Mouser NL	Electronics
EE13	DC Power Connector	1 pcs	0.81	0.81	Mouser NL	Electronics
EE14	Rocker Switch	1 pcs	0.95	0.95	Mouser NL	Electronics
EE15	Wire, Jumper	1 pack	3.67	3.67	Mouser NL	Electronics
EE16	Wire, 20 AWG	5 ft	0.68	3.40	Mouser NL	Electronics
EE17	Wire, 20 AWG	5 ft	0.68	3.40	Mouser NL	Electronics
EE18	Pin Terminals	10 pcs	0.21	2.10	Mouser NL	Electronics
EE19	Breakaway Header	1 pcs	1.40	1.40	Mouser NL	Electronics
EE20	Heat Shrink, 2 mm	1 pcs	0.33	0.33	Mouser NL	Polyolefin
EE21	Heat Shrink, 5 mm	1 pcs	0.52	0.52	Mouser NL	Polyolefin
EE22	DC Adapter, Male	1 pcs	1.77	1.77	Mouser NL	Electronics
EE23	DC Adapter, Female	1 pcs	1.77	1.77	Mouser NL	Electronics
Total Cost **− €**				237.82

**Table 3:** Bill of materials of the WOCA Canister.

**Designator**	**Component**	**Number**	**Cost per unit- €**	**Total cost − €**	**Source of materials**	**Material type**
Manufactured Parts						
MFG05	Canister Lid	1 pcs	N/A	N/A	TU Delft Workshop	PET-G
MFG06	Ball Cage	1 pcs	N/A	N/A		PET-G
MFG07	Piston	1 pcs	N/A	N/A		PET-G
Procured Parts						
ME10	Tubes, OD 6 mm	0.5 *m*	0.51	0.26	Distrelec NL	PU
ME23	3D Printer Filament	94 g	0.06	5.17	RS NL	PET-G
CS01	500 ml Bottle	1 pcs	19.50	19.50	Eurofysica NL	Glass
CS02	Syringe, 10 ml	1 pcs	0.56	0.56	24Pharma NL	Polymer
CS03	Syringe, 5 ml	1 pcs	0.59	0.59	24Pharma NL	Polymer
CS04	Bacterial Filter	1 pcs	5.31	5.31	AliExpress	Polymer
CS05	Hose Bulkhead Connector	1 pcs	0.89	0.89	AliExpress	Polymer
CS06	Syringe Adapter	1 pcs	0.36	0.36	Amazon NL	Polymer
CS07	Table Tennis Ball	1 pcs	0.41	0.41	Decathlon NL	Polymer
CS08	Silicone Gasket	1 pcs	3.50	3.50	Local Hardwarehttps://nl.rs-online.com/web/p/gaskets-o-rings/2556018?gb=s	Silicone
CS09	Quick Glue	1 pcs	4.99	4.99	Amazon NL	Polymer
Total Cost **− €**				41.54		

## 5 Build instructions

### 5.1 Vacuum pump assembly

#### 5.1.1 Prepare Equipment

The equipment listed in Table 4 are needed during the assembly of the vacuum pump.

**Table 4:** Equipment needed to build the WOCA vacuum pump.

**No.**	**Equipment**	**Model Used**
1	3D Printer	Ultimaker S2+
2	Multimeter	RS PRO IDM 71
3	Wire Stripper	RS PRO 613-044
4	Ferrule Crimp Tool	RS PRO 122-1790
5	Side Cutter	RS PRO 536-420
6	Soldering Station	Weller Digital Rework Station WXR3 (solder & heat gun)
7	Hex Key 2.5 mm	N/A
8	Hex Key 3.0 mm	N/A
9	Screwdriver 2.5 mm	N/A
10	Laptop Computer	Macbook Pro

#### 5.1.2 3D print parts

Download STL files provided in the design file folder. Import the STL files to the preferred 3D printer slicing software, e.g. Cura (Ultimaker, the Netherlands) for slicing. A layer height of 0.2 mm, 20 % infill density, and a tree support only on build plate is recommended. Save file in Gcode file format and import to the 3D printer to start printing. Wait for 3D print to finish. Remove all support and file the burred edges on the finished parts. A hot air gun can be used optionally to clean up the parts.

**Fig. 8:**
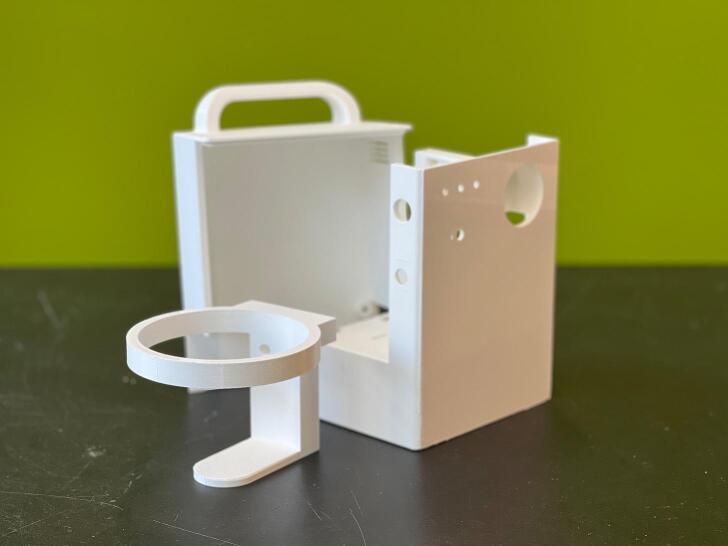
3D printed parts of the WOCA Vacuum Pump.

PET-G is the preferred choice of material due to its higher shock resistance and chemical stability. Other common materials such as PLA and ABS can also be used depending on availability. Fig. 8 shows the 3D printed parts.

#### 5.1.3 Solder wires to electronic components

**Fig. 9:**
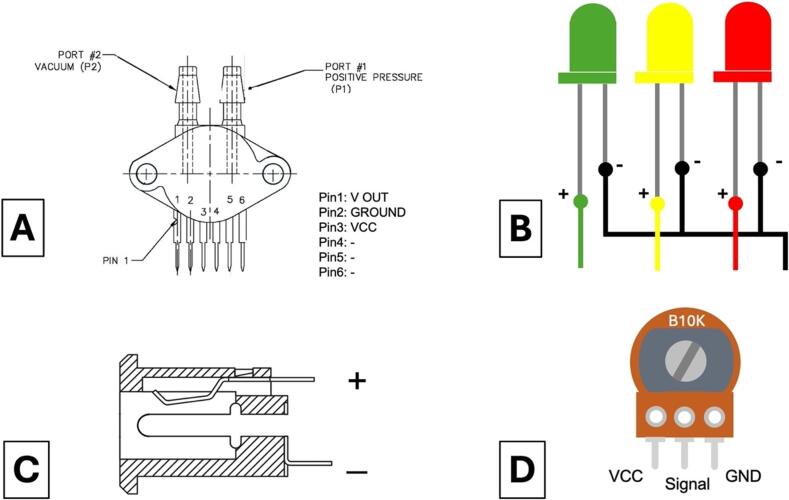
Connection terminals placements for electrical components. (A) Pressure sensor (B) LEDs (C) DC power connector (D) Potentiometer.

**Fig. 10:**
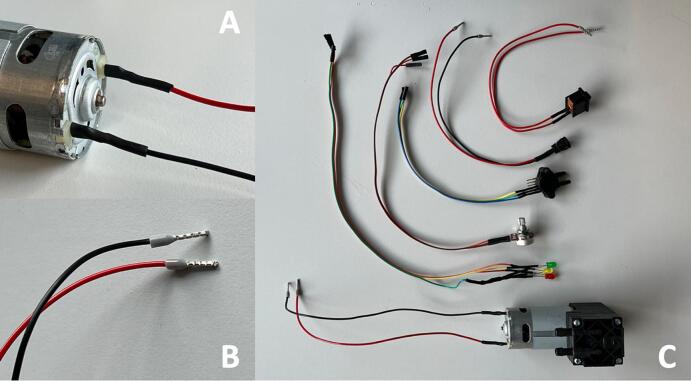
(A) Heat shrinks applied to protect solder. (B) Pin terminals connections. (C) A display of all components soldered with wires.

The components that needed to have wires soldered are listed in the Table 5. Cut wires to lengths as specified in the table and strip ends with a wire stripper to solder. Use red AWG 20 wires (EE16) for live connections and black AWG 20 (EE17) for neutral connections. Use the ferrule crimp tool to make the connectors for the pin terminals (EE18). For the jumper wires, there is no need to make the female Dupont connectors. The jumper wires (EE15) should come with both ends already in Dupont typed connectors. Simply cut one end to length and solder the wires to the components. Refer to Fig. 9 for guidance on connector terminal placements for the electrical components. Use heat shrinks to protect the solder (Fig. 10A). All components soldered with wires are shown in Fig. 10C.

**Table 5:** List of components needed to have wires soldered.

**Designator**	**Component**	**Wire Type**	**Length**	**Connector Type**
ME01	Vacuum Pump	AWG 20 Wire (Red & Black)	20 cm	Pin Terminals
EE03	Pressure Sensor	Jumper Wire	20 cm	Female Dupont
EE04	Potentiometer	Jumper Wire	25 cm	Female Dupont
EE13	DC Power Connector	AWG 20 Wire (Red & Black)	20 cm	Pin Terminals
EE14	Rocker Switch	AWG 20 Wire (Red)	20 cm	Pin Terminals
EE07-09	LED Red, Green, Yellow	Jumper Wire	30 cm	Female Dupont

#### 5.1.4 Solder electronic components to Prototype Board

**Fig. 11:**
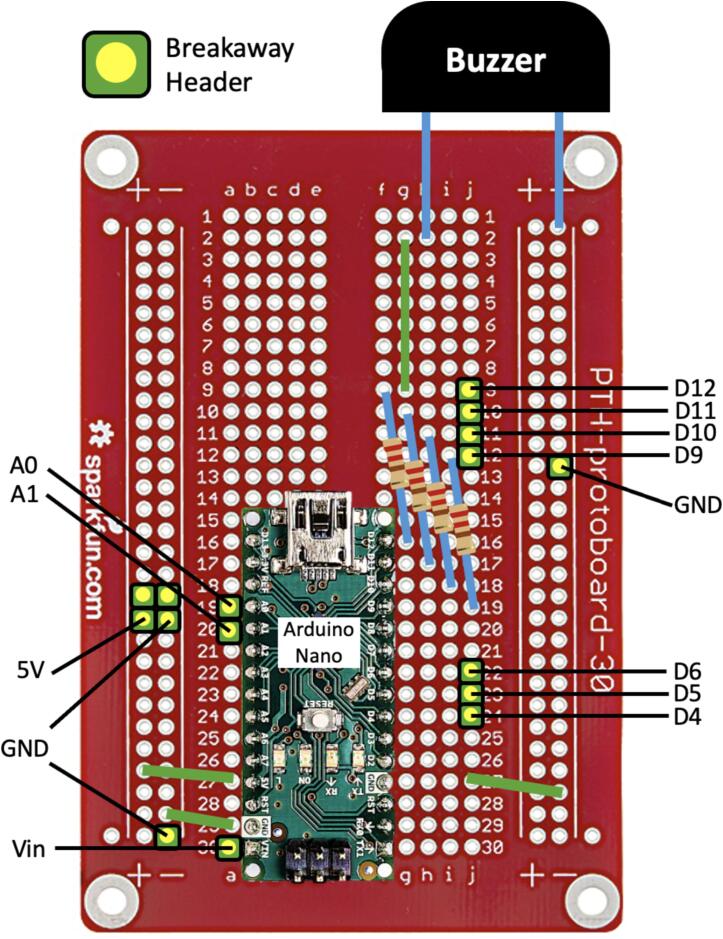
Schematic of the connections on the prototype board.

A schematic of the connections on the Prototype Board (EE12) are shown in Fig. 11. The Piezo Buzzer (EE10), Microcontroller (EE01) and Resistors (EE06) are soldered on the board. Cut AWG20 wires (EE16) to make connections on the board. Cut and solder Breakaway Headers (EE19) to make the pin connectors. Check solders with a multimeter.

#### 5.1.5 Assemble components to Hole Grid

**Fig. 12:**
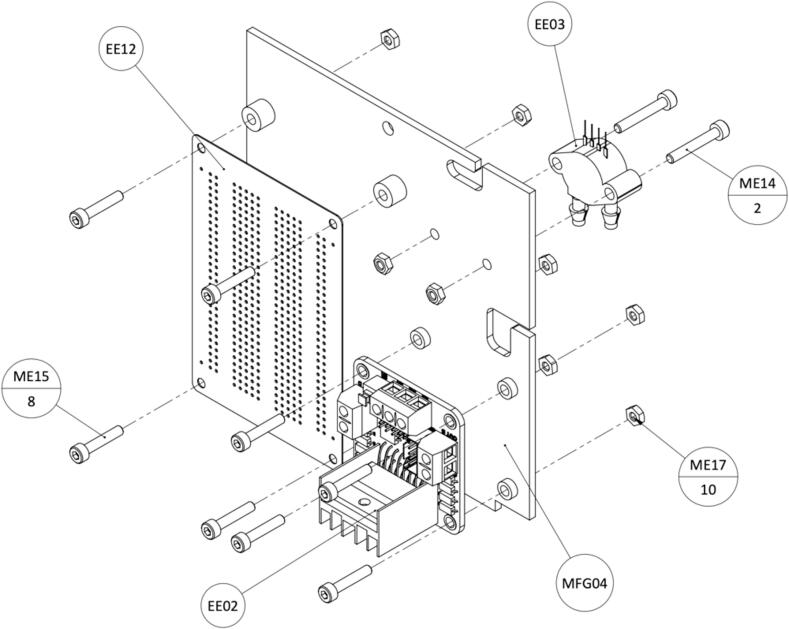
Instructions for assembling components on the Hole Grid.

**Fig. 13:**
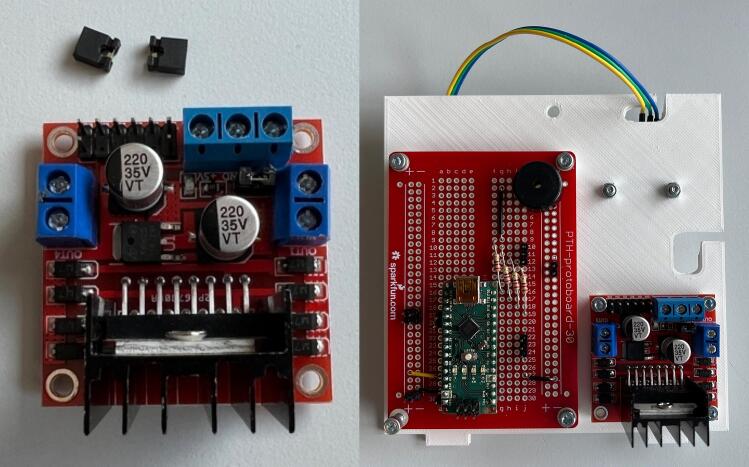
(Left) Motor Driver with jumper pins removed. (Right) Components assembled on the Hole Grid.

Assemble the soldered Prototype Board (EE12), Motor Driver (EE02), and Pressure Sensor (EE03) to the Hole Grid (MFG04) as shown in Fig. 12. Remove jumper pins (ENA, ENB) on the motor driver (Fig. 13).

#### 5.1.6 Install pressure gauge

**Fig. 14:**
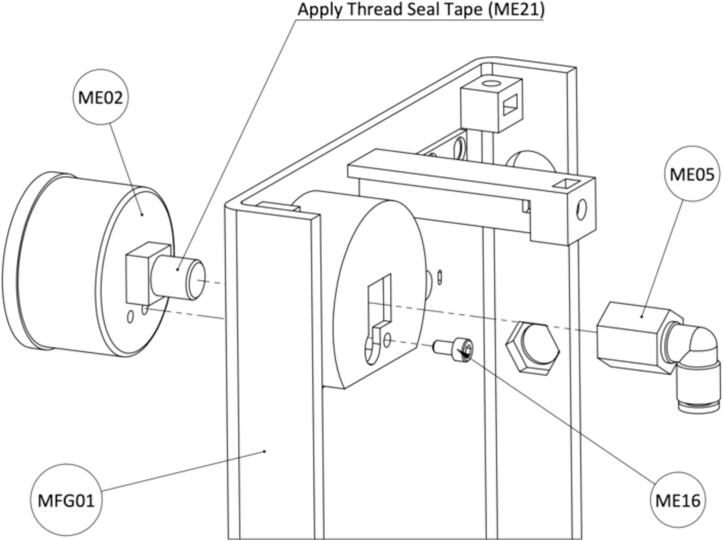
Instructions for installing the Pressure Gauge on the Front Housing.

Install the Pressure Gauge (ME02) to Front Housing (MFG01) as shown in Fig. 14. Remove the right screw from the pressure gauge. Insert the pressure gauge into the slot and screw it to the housing with a M3x6mm Bolt (ME16). Apply Thread Seal Tape (ME21) on the thread when assembling the Elbow Female Connector (EE05).

#### 5.1.7 Assemble components to front housing

**Fig. 15:**
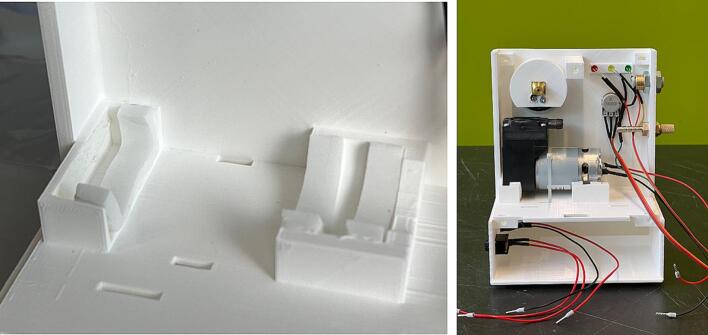
(Left) Foam Tape applied to the housing for the vacuum pump. (Right) Components assembled on the Front Housing.

**Fig. 16:**
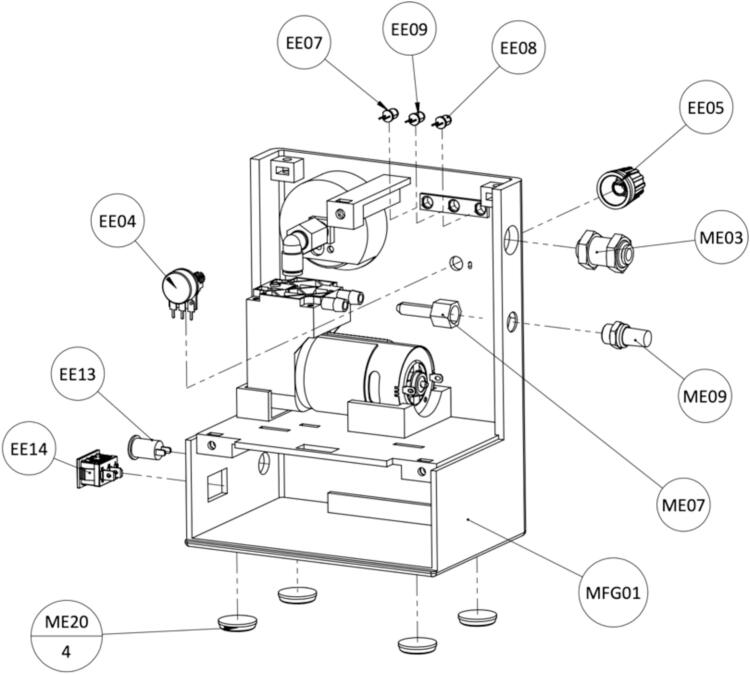
Instructions for assembling components to the Front Housing.

Cut strips of Foam Tape and apply to housing for the vacuum pump as shown in Fig. 15. Slide the Vacuum Pump (ME01) in and secure with Cable Ties (ME19). Assemble the rest of the components to the housing as shown in Fig. 16.

#### 5.1.8 Connect tubing

**Fig. 17:**
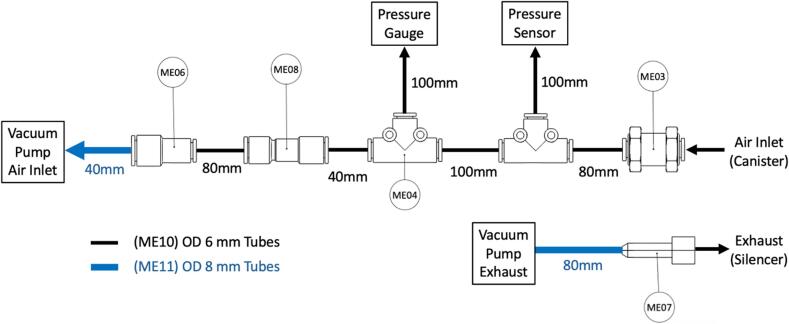
Instructions for connecting the tubing for the WOCA vacuum pump.

**Fig. 18:**
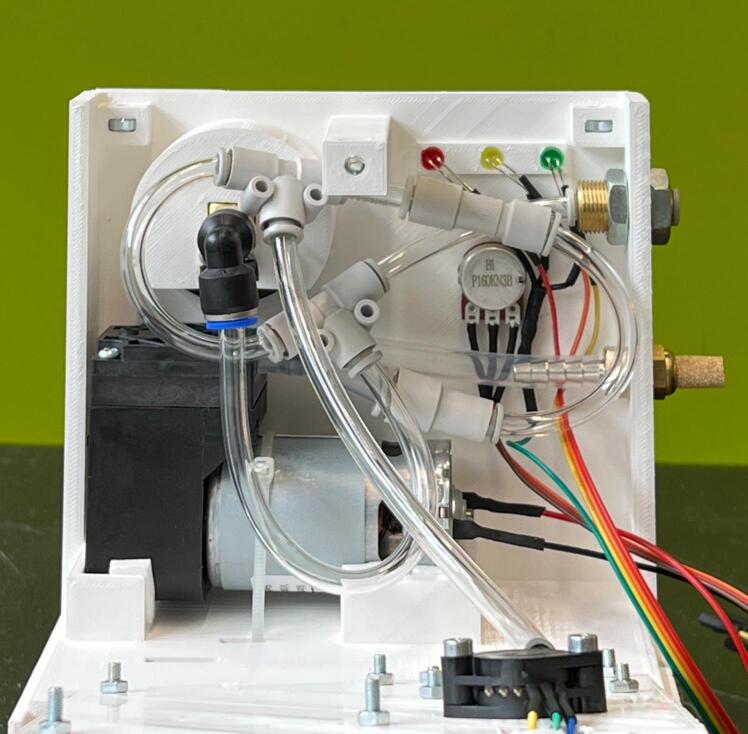
Components in the WOCA vacuum pump with all the tubing connected.

Connect tubing as shown in Fig. 17. OD 8 mm Tube (ME11) are used to make connections with the Vacuum Pump (ME01), otherwise OD 6 mm Tubes (ME10) are used. Cut the lengths of the tubes as specified in Fig. 17. Carefully bend the tubes in a loop to fit into the space inside the Front Housing as shown in Fig. 18.

#### 5.1.9 Complete the electrical circuit connections

Follow the schematic in Fig. 5 to connect the components to the correct input and output pins on the Prototype Board. Use a jumper wire (EE15) to connect pins (ENA, IN1, IN2) on the Motor Driver to pins (D6, D5, D4) on the Prototype Board.

12 V power is supplied from the Lithium Battery to the Motor Driver. The Motor Driver then supplies 5 V power to the Microcontroller. There will be a voltage drop between the 5 V power supply from the Motor Driver and the Microcontroller. It should be noted that the Pressure Sensor and Potentiometer should be wired to the 5 V power supply from the Microcontroller to give correct readings.

**Fig. 19:**
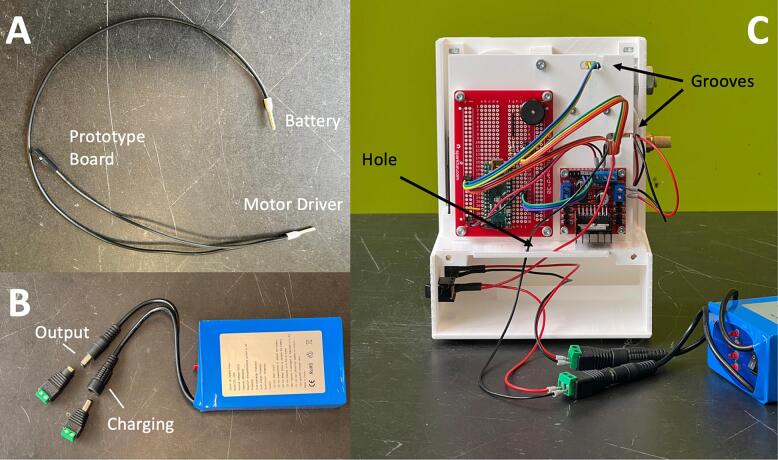
(A) Split wire for the power connections. (B) DC Adapters for the Lithium Battery. (C) Grooves on the Hole Grid and the hole on the Front Housing to protect the wire connections from clipping.

The Lithium Battery (EE11), Prototype Board (EE12), and the Motor Driver (EE02) should share a common ground. Make a split wire connection with a crimped pin terminal (EE18) at the common end and a Female Dupont connector and a crimped pin terminal at the split end. The split wire is shown in Fig. 19A. The common end is connected to the pin (GND) on the Motor Driver. The terminal pin end of the split wire is connected to the cathode of the battery, while the Dupont end is connected to the pin (GND) on the Prototype Board. Use a Male DC Adapter (EE22) for the charging port and a Female DC Adapter (EE23) for the output port of the Lithium Battery. Complete the power connections by wiring the DC Power Connector (EE13) and the Rocker Switch (EE14) to the Lithium Battery (EE11) and Motor Driver (EE02). A screwdriver will be needed to connect the pin terminals.

Insert the wires of the components to the grooves on the Hole Grid. Insert the power connections from the battery compartment through the hole on the Front Housing. These features protect cables from clipping when closing the housing. The completed connection is shown in Fig. 19C.

Turn on the power switch to check if circuit is complete. LED lights of Microcontroller and Motor Driver should light up if the circuit is correctly connected. Use a multimeter to measure voltage to check for connections.

#### 5.1.10 Upload Arduino software

**Fig. 20:**
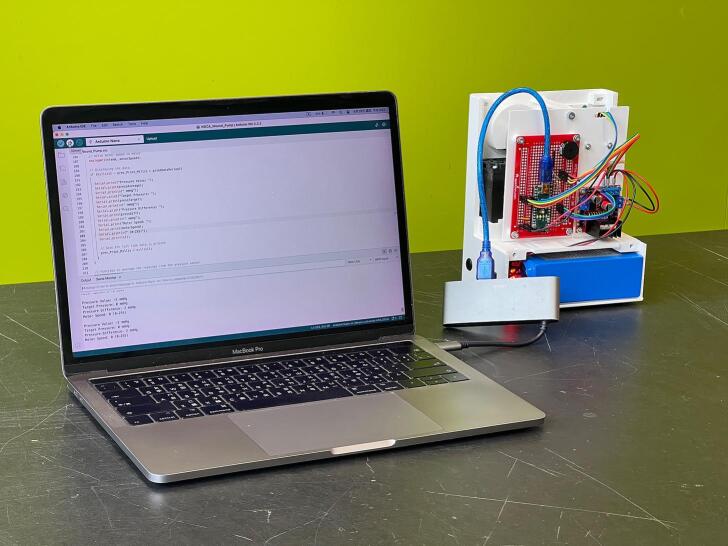
Microcontroller (Arduino Nano) connected to a computer with a USB wire.

Download and install the latest Arduino software (IDE) from the official Arduino website (ww.arduino.cc). Install the environment on a computer. Download the INO file, WOCA Arduino Code (SW01), from the design files folder. Connect the computer and microcontroller with a USB wire (Fig. 20). Upload the code to the microcontroller. Once the upload is complete, the pressure reading values should be visible on the serial communication window in the Arduino IDE software.

#### 5.1.11 Complete the assembly

**Fig. 21:**
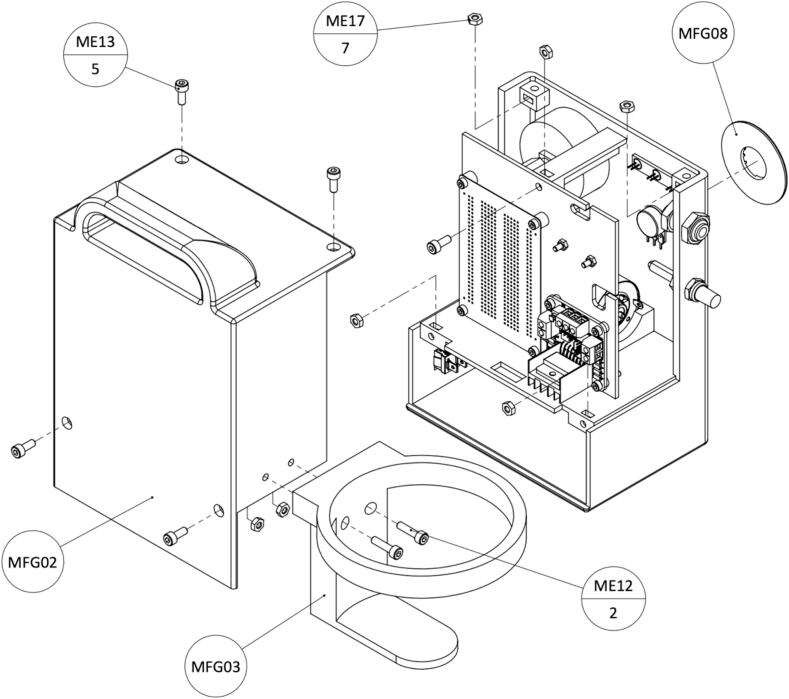
Instructions to assemble Back Housing and Canister Holder.

**Fig. 22:**
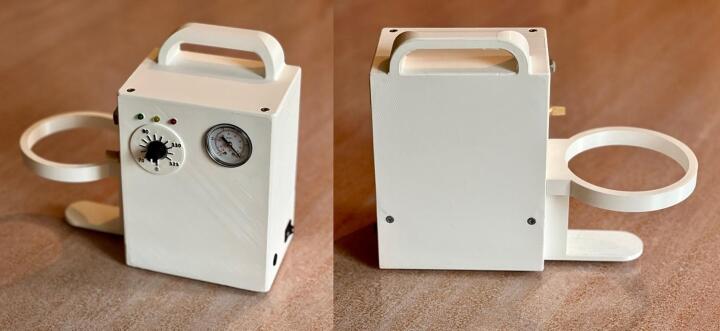
Completed WOCA Vacuum Pump.

Complete the assembly as shown in Fig. 21. Assemble Back Housing (MFG02) and the Canister Holder (MFG03). Insert the nuts in the grooves of the Front Housing (MFG01). Screw the bolt on the Hole Grid (MFG04). Close the Back Housing and screw the rest of the bolts. Finally, use quick glue to attach the Pressure Setting Plate (MFG08) to finish the assembly. Turn the control knob counterclockwise to the end. The indicator should align to the pressure setting zero. The completed WOCA Vacuum Pump is shown in Fig. 22.

### 5.2 Canister assembly

#### 5.2.1 Prepare Equipment

The equipment listed in Table 6 are needed during the assembly of the WOCA canister.

**Table 6:** Equipment needed to build the WOCA canister.

**No.**	**Equipment**	**Model Used**
1	3D Printer	Ultimaker S2+
2	Side Cutter	RS PRO 536–420

#### 5.2.2 3D print parts

**Fig. 23:**
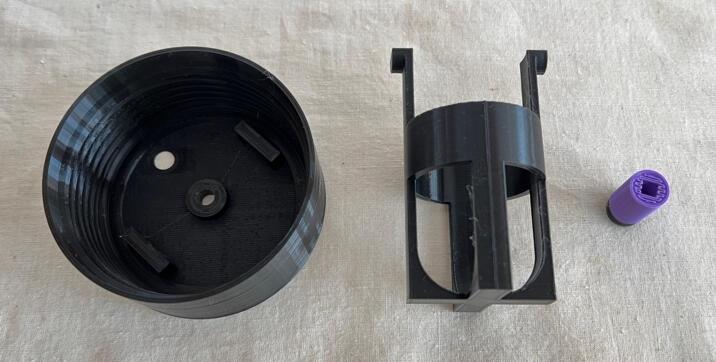
3D printed parts of the WOCA Canister.

Download STL files provided in the design file folder and 3D print as described in assembly in vacuum pump. Use a finer setting (layer height: 0.16 mm) and a higher minimum wall/perimeter line count (wall line count: 4) to print the canister lid (MFG05) to ensure airtight performance. For the ball cage (MFG06) and the piston (MFG07), normal settings can be used. Fig. 23 shows the printed parts.

#### 5.2.3 Assemble canister lid

**Fig. 24:**
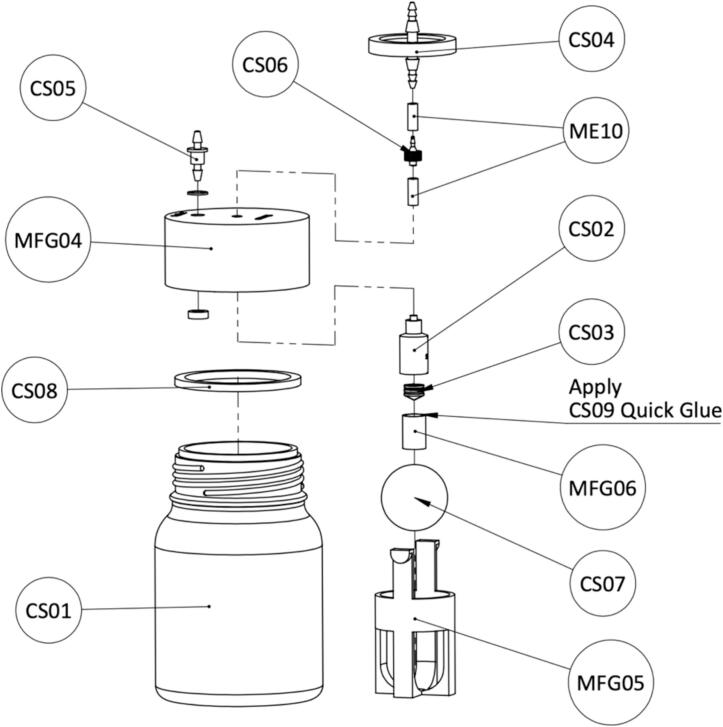
Instructions for assembling the WOCA canister lid.

**Fig. 25:**
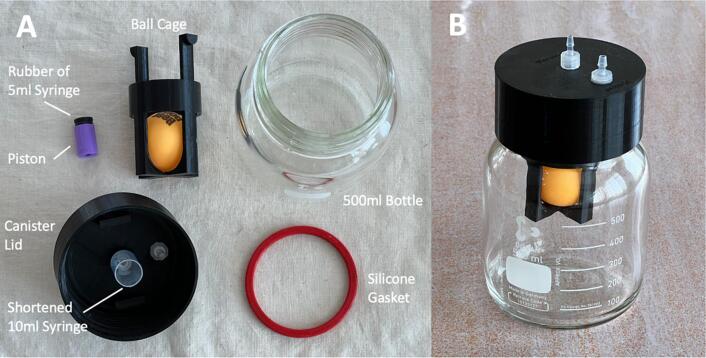
(A) Components of WOCA Canister. (B) WOCA Canister after assembly.

Assemble the canister as shown in the exploded view in Fig. 24. The rubber seal is removed from of a 5 ml syringe (CS03) and glued with the piston (MFG06) using quick glue (CS09). A 10 ml syringe is cut to length (35 mm) to serve as a shaft for the piston. A short OD 6 mm Tube (ME10) with a length of 16 mm is inserted to the opening to the vacuum pump on the canister lid (MFG04). There might be some tolerance on the tube and 3D printed canister lid opening. A tight fit is expected to make the lid airtight. In case of difficulty in inserting the tube, a round file can be used to carefully widen the opening. An in-line bacterial filter is connected to the opening of the vacuum pump with another OD 6 mm Tube (ME10). Take note of the direction of the filter. The air inlet of the filter should be facing towards the canister lid and outlet should be connected to the vacuum pump. Fig. 25 shows the complete canister before and after the assembly.

## 6 Operation instructions

### 6.1 Description of operation

**Fig. 26:**
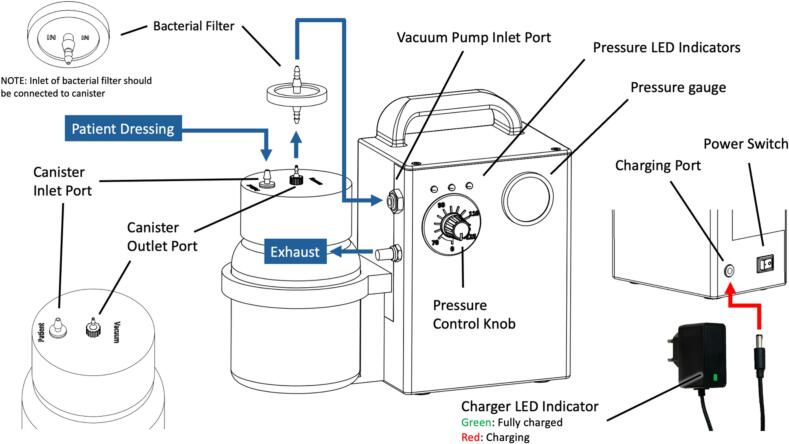
Connections and controls of the WOCA Pump System.

The connections and controls of the WOCA Pump are illustrated in Fig. 26. Air and exudate (body fluid) from the patient dressing enters the system via the inlet port of the canister. The body fluids are stored in the canister while air exits via the outlet port of the canister. Air exiting the outlet port of the canister is filtered and enters the inlet port of the vacuum pump. Vacuum pressure is generated by a vacuum motor inside the pump and regulated by the microcontroller. The pressure gauge and pressure sensor sense the vacuum pressure generated and sustained throughout the vacuum pump, canister, and dressing pad. Exhaust air of the vacuum motor passes through a silencer before exiting the vacuum pump.

The power of the device can be turned on and off by a power switch. Pressure settings are adjusted through a control knob from a range of 75 to 125 mmHg. A 12 V 6800 mAh rechargeable battery is installed inside to supply power. Battery capacity is designed to sustain continuous mobile operation of the system throughout at least a single treatment (3–5 days). A 12 V DC charger can be connected to the charging port to charge the battery. LED indicator on the charger shows red when charging and green when fully charged.

**Fig. 27:**
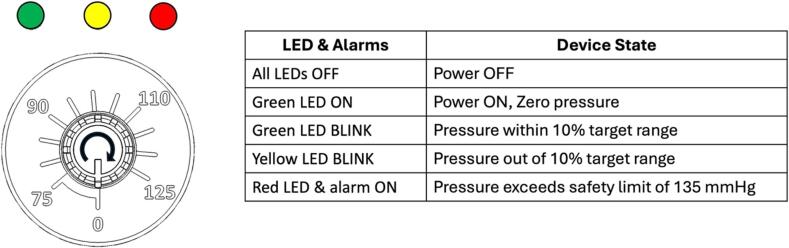
Definition of different LEDs and alarm states in the WOCA Vacuum Pump. Rotate the control knob clockwise to adjust target pressure. Pressure range from 75 to 125 mmHg.

The definitions of the pressure LED indicators and alarms are shown in Fig. 27. The LEDs, buzzer alarm, and pressure gauge are used as indicators for the device. When the power is switched on, there will always be two short blink/ beep on all the LEDs and the buzzer. The green LED will be constantly on when the when pressure setting is zero. The pressure knob is turned clockwise and adjusted to a targeted pressure and the vacuum pump will start to operate. The yellow LED will start to blink when pressure is out of range. The pump will continuously operate until the system reaches the desired pressure range (within 10 % of targeted pressure). Once pressure reaches the settings, the green LED will start to blink. The vacuum pump will operate intermittently to maintain the pressure in the system. The pressure gauge can be used to verify whether the system is operating in the desired pressure settings.

A safety limit is set to the system to protect the device from damage and patient from harm. The red LED and buzzer alarm will be constantly on when vacuum pressure exceeds 135 mmHg. The motor will also be forced to stop until the vacuum pressure of the system resumes under the safety limit.

There is also an overflow protection valve on the canister. Once the body fluid reaches the maximum capacity that can be stored, it triggers the overflow valve to block the air inlet of the vacuum pump. The alarm of the vacuum pump will set off and the motor will stop operating until the canister is emptied.

### 6.2 Guideline for pressure setting

The recommended pressure level for NPWT is 125 mmHg, which is considered the optimum pressure for most wounds [4], [5], [6]. Research indicates that the effective range for this treatment lies between 75 and 125 mmHg [4]. In specific situations, reduced pressure is preferred, for example when treating sensitive wounds or when the patient experiences pain during treatment [4]. A pressure level of 80 mmHg is known to be used in clinical practice, mostly when treating smaller or sensitive wounds. It was found that when applying 80 mmHg granulation is still formed, while below 75 mmHg the effect significantly decreases [3], [4]. The maximum acceptable pressure is 200 mmHg, because stronger pressures no longer stimulate healing and can result in tissue damage [4].

### 6.3 Standard operation procedure

The standard operation procedure for clinical use are shown in Table 7. Please follow the steps and read the warnings.

**Table 7:** Standard operation procedure for clinical use.

**Step**	**Instruction**	**Description**
1	Charge Battery	It usually needs > 8hr to fully charge battery. The indicator on the charger turns red when charging and turns green when fully charged. Currently, the device has no battery level indicator function yet.
2	Connect Vacuum Pump and Canister	Take note of the markings on the canister lid. Connect the inlet port of the vacuum pump to the filter and to the outlet port of the canister (marked with “Vacuum”). **WARNING:** Do not connect reverse connections of the inlet and outlet port of the canister. This may result in the overflow valve to not function and damage the vacuum pump.
3	Turn Power ON	Check if control knob is turned to 0. Turn on the power switch and look at the LEDs and listen to the buzzer. There should be two short blinks and two short beeps once the power is turned on.
4	Check Pressure	Check if the green LED is constantly lighted while the pressure setting is set to zero. Readings on the pressure gauge should also be zero.
5	Adjust Pressure Setting	Turn the control knob clockwise slowly and listen if the motor is operating. Block the patient outlet on the canister and set the dial to 125 mmHg. The yellow LED should blink constantly while the motor is operating. Once vacuum pressure reaches the adjusted setting, the green LED will start to blink.
6	System Check	• Monitor that the vacuum pressure reaches the target pressure set by the dial and then switches off within target pressure + 10 % and the status LED turns green. Monitor that pump stays off for at least 2 min. Monitor that the pump switches on again when reaching 10 % under the target pressure. While the pump is running squeeze the tube between pump and canister. The red LED and an audible alarm should come immediately, and the pump should stop. If any of the above is not happening as expected check all connections for leakage. If this does not resolve the issue then the device needs inspection in the technical department. Switch the device off and proceed to connect to the patient.
7	Connect Dressing Pad	Connect dressing pad to the inlet port of the canister (marked with “Patient”). **WARNING:** Do not connect the dressing pad directly to the inlet port of the vacuum pump. This may harm the patient and damage the device. A canister with filter should always be used.
8	Treatment	Switch on the pump and set the correct vacuum pressure. Monitor the status LED becomes green and the pump is switched of for at least 2 min before starting again. Check for dressing leakage if the behavior is different. Leave the power of the vacuum pump on during the treatment. The green LED should continue to blink throughout the treatment. The microcontroller will control the vacuum pump to regulate the pressure.
9	Ending Treatment	Turn the control knob counterclockwise until pressure setting is 0. The yellow LED will constantly blink due to the residual pressure inside the system. The vacuum pump will stop operating and pressure will gradually drop. Turn power switch off.
10	Disconnect Vacuum Pump, Canister, and Dressing Pad	Disconnect the dressing pad from the canister. There will be a sudden drop of readings on the pressure gauge due to the release of pressure. Disconnect the vacuum pump and canister. **WARNING:** Do not disconnect the system while the power is ON.
11	Cleaning	Dispose the exudate stored in the canister properly. Disassemble and clean the canister before use again, according to the hospital’s cleaning and disinfection procedures.

### 6.4 User maintenance procedure

#### 6.4.1 Cleaning and disinfection

It is recommended to clean and disinfect the WOCA:

• If it becomes soiled during patient use.
• At least weekly.

Follow the procedures shown in Table 8.

**Table 8:** Cleaning and disinfection procedure of WOCA.

**Step**	**Instruction**	**Description**
1	Turn off Power	Ensure the power switch of WOCA is turned off (LED lights all dimmed) and charger is disconnected.
2	Disconnect Vacuum Pump and disassemble canister	Disconnect tubing connecting the vacuum pump and canister. Remove and disassemble canister.
3	Clean Organic Material	Wipe down all hard surface components. Clean all visible soil or body secretion from prior to disinfection. **WARNING:** Use hospital grade cleaners and disinfectants. **WARNING:** Avoid using alcohol based solutions around power switches and electronic components.
4	Disinfect Vacuum Pump and canister	Follow institutional procedures used for disinfection. Use a damp cloth to disinfect the Vacuum Pump. **WARNING:** Do not immerse or saturate Vacuum Pump with fluids to avoid damage to the electronics in the device.
5	Dry and assemble device	Make sure the device is dry before tuning on the power. Follow instruction in Section 5.2 to reassemble canister.

## 7 Validation and characterization

### 7.1 Leakage test

**Fig. 28:**
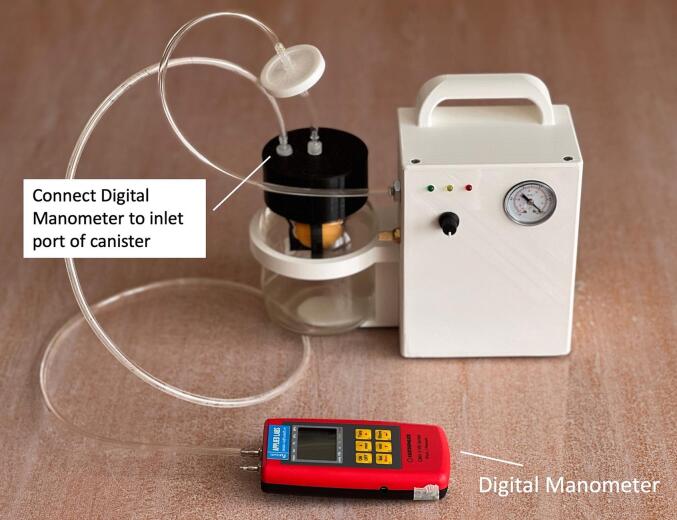
Setup for leakage test of the WOCA Pump.

A leakage test (Table 9, Table 10, Fig. 28) is performed to verify whether leaks in the system (vacuum pump and canister) is acceptable. The pressure difference between measurements in 0 min and 3 min should be less than 10 mmHg.

#### 7.1.1 Equipment

**Table 9:** Equipment used to perform the WOCA Pump leakage test.

**No.**	**Equipment**	**Model used**
1	Timer	Iphone timer function
2	Handheld Digital Manometer	Greisinger GMH 3100

#### 7.1.2 Test instructions

**Table 10:** Instructions for the WOCA Pump leakage test.

**Steps**	**Instruction**	**Description**
1	Connect Vacuum Pump to Canister	Connect the inlet port of the vacuum pump to the bacterial filter and outlet port of the canister (marked with “Vacuum”).
2	Connect Handheld Digital Manometer	Use a PU tube (OD 6 mm, ID 4 mm) and connect the handheld digital manometer to the inlet port of the canister (marked with “Patient”).
3	Turn Power ON	Check if control knob is turned to 0. Turn on the power switch.
4	Adjust Pressure	Turn the control knob clockwise to adjust pressure settings to 125 mmHg.
5	Wait for Pressure to Increase	The pressure is expected to gradually increase while the yellow LED continue to blink. When pressure reaches 125 mmHg, the green LED will start to blink.
6	Turn Power OFF	Wait for around 3 min for pressure to stabilize and then turn the power switch off.
7	Record Pressure Measurements	Start timer and record pressure measurements from the handheld digital manometer (0 min, 1 min, 2 min, 3 min, 5 min).

#### 7.1.3 Test results

Test results of the leakage test are shown in Table 11. With a pressure setting of 125 mmHg, measured pressure within the system from 0 to 3 min dropped from 123 mmHg to 117 mmHg. A 6 mmHg drop of pressure shows minimal leakage for the system and is considered acceptable.

**Table 11:** Results for the WOCA Pump leakage test.

Pressure Setting	Handheld Digital Manometer Measurement				
	0 min	1 min	2 min	3 min	5 min
125 mmHg	123	122	120	117	112

### 7.2 Pressure control test

A pressure control test (Table 12) is performed to verify how well pressure is controlled. The vacuum pump is connected to the canister and adjusted to different pressure settings (Table 13). Readings from the digital manometer, pressure gauge, and the pressure sensor should all be within 10 % range of pressure settings.

#### 7.2.1 Equipment

**Table 12:** Equipment used to perform the WOCA Pump pressure control test.

**No.**	**Equipment**	**Model used**
1	Timer	Iphone timer function
2	Handheld Digital Manometer	Greisinger GMH 3100
3	Laptop Computer	Macbook Pro

#### 7.2.2 Test instructions

**Table 13:** Instructions for the WOCA Pump pressure control test.

**Steps**	**Instruction**	**Description**
1	Connect Computer	Remove Back Housing. Use a usb cable to connect the Arduino microcontroller to the computer. Open the serial monitor in Arduino IDE to read sensor values.
2	Connect Vacuum Pump to Canister	Connect the inlet port of the vacuum pump to the filter (outlet port) of the canister.
3	Connect Handheld Digital Manometer	Use a PU Tube (OD 6 mm, ID 4 mm) to connect the handheld digital manometer to the suction inlet of the canister.
4	Turn Power ON	Check if control knob is turned to 0. Turn on the power switch.
5	Adjust Pressure	Turn the control knob clockwise to adjust pressure settings to 75, 90, 110, and 125 mmHg.
6	Wait for Pressure to Increase	The pressure is expected to gradually increase while the yellow LED continue to blink. When pressure reaches 125 mmHg, the green LED will start to blink.
7	Record Pressure Measurements	Start timer and record pressure measurements from the handheld digital manometer and the pressure gauge at 0, 3, 5, and 10 min.
8	Turn Power OFF	Turn the control knob counterclockwise to 0 and turn off the power switch. Disconnect the digital manometer.

#### 7.2.3 Test results

**Fig. 29:**
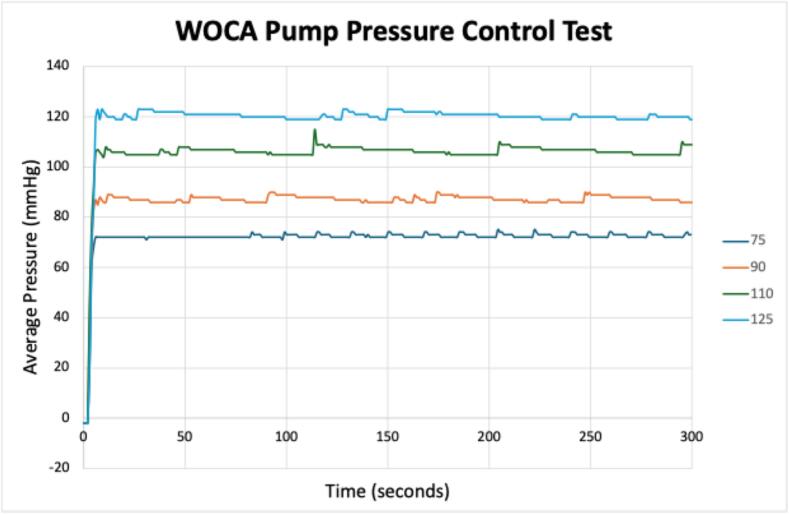
Recorded pressure values from the sensor in the WOCA Pump pressure control test.

Test results for the pressure control tests are shown in Table 14 and Fig. 29. These results show that the pressure control is stable within the 5-minute testing time frame. Small spikes are visible In the values recorded from the sensor, showing that the motor is turned on to compensate the loosing pressure due to air leakage.

**Table 14:** Recorded pressure values from the digital manometer and pressure gauge in the WOCA Pump pressure control test.

Pressure Setting (mmHg)	Measurement Device	Pressure Measurement in Different Time		
		0 min	3 min	5 min
75	Digital Manometer	76	78	77
	Pressure Gauge	75	75	75
90	Digital Manometer	92	90	91
	Pressure Gauge	88	88	88
110	Digital Manometer	111	110	111
	Pressure Gauge	110	110	110
125	Digital Manometer	125	124	123
	Pressure Gauge	123	120	120

### 7.3 Safety alarm test

A safety alarm test (Table 15) is performed to verify whether the safety mechanisms of the device are functioning. For this test, no extra equipment is needed. The canister is disconnected from the vacuum pump. A sudden blockage of the air inlet of the vacuum pump will cause a sudden increase of pressure within the system. This should light the red LED, sound the buzzer alarm, and stop the motor. The test is repeated three times for repeatability.

#### 7.3.1 Test Instructions

**Table 15:** Instructions for the WOCA Pump safety alarm test.

**Steps**	**Instruction**	**Description**
1	Disconnect Canister	Disconnect tubing between vacuum pump and canister.
2	Turn Power ON	Check if control knob is turned to 0. Turn on the power switch.
3	Set Pressure to 125 mmHg	Turn the control knob clockwise to adjust pressure setting to 125 mmHg. The vacuum pump will operate continuously.
4	Block Vacuum Pump Air Inlet	Use a finger or a tube cap to block the tube from the vacuum pump. The pressure is expected to rise sharply and set off the safety alarm.
5	Release Pressure and Turn Power Off	Turn the control knob to 0. Release the finger from the tube to release the pressure. Turn off the power of the device after testing.

### 7.4 Overflow valve test

An overflow valve test is performed to verify whether the device will stop when the canister is full. Only an additional water container that is at least 500 ml is needed to perform this test. When the canister is full, the overflow valve will block the air passage to the vacuum pump. The sudden spike in pressure will trigger the safety alarm and stop the motor. The test is repeated three times for repeatability. When overflow valve is not functioning water will pass the valve and travel to the pump quickly. Make sure to shut down the pump immediately to prevent fluid from entering the device.

#### 7.4.1 Test Instructions

**Table:** 

**Steps**	**Instruction**	**Description**
1	Fill Container	Fill water container with water.
2	Connect Vacuum Pump to Canister	Connect the inlet port of the vacuum pump to the filter (outlet port) of the canister.
3	Put Suction Tube in Water Container	Use a PU Tube (OD 6 mm, ID 4 mm) to connect the suction inlet of the canister. Emerge the opening of the tube in the water.
4	Turn Power ON	Check if control knob is turned to 0. Turn on the power switch.
5	Set Pressure to 125 mmHg	Turn the control knob clockwise to adjust pressure setting to 125 mmHg.
6	Wait for Canister to Fill	Water from the container will start filling the canister. Once maximum capacity of the canister is reached, the overflow valve should be triggered.
7	Turn Power OFF and Empty Canister	Turn control knob to 0 and turn power off. Remove the tube from the water. Open the lid of the canister and empty the water.

### 7.5 Preventive maintenance

It is advised to repeat the following test at least once a year:

• Device leakage test: refer to procedures described in Chapter 7.1.
• Pressure calibration: refer to procedures described in Chapter 7.2.
• Safety alarm test: refer to procedures described in Chapter 7.3.
• Overflow alarm test: refer to procedures described in Chapter 7.4.

To ensure hygiene and protect the vacuum pump, it is recommended to replace the bacterial filter (CS04) at least every two months. However, the filter should be replaced immediately if any of the following occurs:

• Overflow of the canister.
• Visible dirt or debris buildup.
• Reduced performance, such as increased pressure at the patient end.

### 7.6 General troubleshooting

**Fig. 30:**
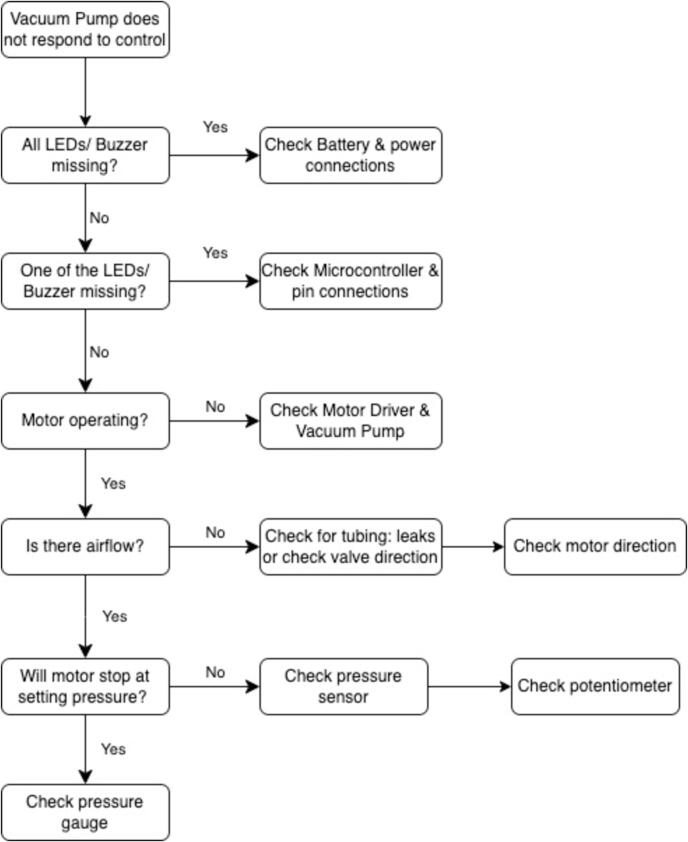
Flowchart for troubleshooting the WOCA.

Before reviewing the troubleshooting chart (Fig. 30), the following steps may be useful to isolate any malfunctions:

1. Make sure the battery is fully charged.
2. Make sure the filter and tubes are clean.
3. Make sure the canister is empty.
4. Make sure there are no leaks by checking the fittings and connectors. Reapply thread seal if there is leakage on the threads. Make a clean cut on the PU tubes or replace the tubes and reconnect to the push-in fittings.

## 8 Ethics statements

No animal or human subjects were used in this study.

## CRediT authorship contribution statement

**Arjan J. Knulst:** Writing – review & editing, Supervision, Resources, Project administration, Methodology, Investigation, Funding acquisition, Conceptualization. **Salome Berger:** Validation, Conceptualization. **Jorijn van den Boom:** Software, Methodology, Investigation. **Inge Bosch:** Software, Methodology, Investigation. **Noa Nicolai:** Software, Methodology, Investigation. **Suraj Maharjan:** Validation, Conceptualization. **Eileen Raaijmakers:** Visualization, Methodology, Investigation, Data curation, Conceptualization. **Chang-Lung Tsai:** Writing – original draft, Visualization, Software, Data curation. **Lisa van de Weerd:** Validation, Methodology, Investigation, Data curation, Conceptualization. **Jenny Dankelman:** Writing – review & editing, Supervision, Funding acquisition, Conceptualization. **Jan-Carel Diehl:** Writing – review & editing, Supervision, Funding acquisition, Conceptualization.

## Declaration of competing interest

The authors declare that they have no known competing financial interests or personal relationships that could have appeared to influence the work reported in this paper.

## References

[1] Järbrink K. (2016). Prevalence and incidence of chronic wounds and related complications: a protocol for a systematic review. Syst. Rev..

[2] Mazuz R. (2019). Wound care in the developing world - gaps, opportunities, and realities. Curr. Dermatol. Rep..

[3] L.C. Argenta, M.J. Morykwas, Vacuum-assisted closure: a new method for wound control and treatment: clinical experience*,* Ann. Plast. Surg. 38 (6) (1997) 563-76; discussion 577. https://doi.org/10.1097/00000637-199706000-00002. 9188971

[4] Apelqvist J. (2017). EWMA document: negative pressure wound therapy. J. Wound Care..

[5] Huang C. (2014). Effect of negative pressure wound therapy on wound healing. Curr Probl Surg..

[6] Yadav S., Rawal G., Baxi M. (2017). Vacuum assisted closure technique: a short review. Pan Afr. Med. J..

[7] Agarwal P., Kukrele R., Sharma D. (2019). Vacuum assisted closure (VAC)/negative pressure wound therapy (NPWT) for difficult wounds: A review. JCOT..

[8] Borgquist O., Ingemansson R., Malmsjö M. (2011). The Influence of low and high pressure levels during negative-pressure wound therapy on wound contraction and fluid evacuation. Plast. Reconstr. Surg..

[9] Braakenburg A. (2006). The clinical efficacy and cost effectiveness of the vacuum-assisted closure technique in the management of acute and chronic wounds: a randomized controlled trial. Plast. Reconstr. Surg..

[10] Kim J.J. (2017). Cost-effective alternative for negative-pressure wound therapy. PRS GO..

[11] E. Raaijmakers, Design of a low-cost device for Negative Pressure Wound Therapy in low and middle-income countries, in Industrial Design Engineering. 2022, Delft University of Technology. https://resolver.tudelft.nl/uuid:35d644fe-900d-4c52-80eb-6b1398250035.

[12] Älgå A. (2022). Cost analysis of negative-pressure wound therapy versus standard treatment of acute conflict-related extremity wounds within a randomized controlled trial. World J. Emerg. Surg..

[13] Fout B., Plotzke M. (2022). Comparing traditional and disposable negative-pressure wound therapy use by medicare home health patients. Adv. Skin Wound Care..

[14] Cocjin H.G.B. (2019). Wound-healing following negative-pressure wound therapy with use of a locally developed AquaVac system as compared with the vacuum-assisted closure (VAC) system. J. Bone Jt. Surg..

[15] N. Nicolai, WOCA A battery powered wound pump designed for use in low-resource settings, in Technical innovations in Medicine Congres. 2023: Utrecht, Netherlands. https://resolver.tudelft.nl/uuid:0461f27c-986a-4962-a674-9b8ba8db9f45.

[16] L. van de Weerd, Towards a Clinical Trial of the WOCA, in Mechanical Engineering. 2024, Delft University of Technology. https://resolver.tudelft.nl/uuid:fe23eaea-d852-4a9f-981f-fd54e4691643.

